# Koch’s “Cure”

**Published:** 1890-12

**Authors:** 


### KOCHS' "CURE."

The all absorbing question not only in medical circles but in the secular press is Koch's "Consumption Cure," and naturally there is great diversity of opinion upon the subject. Many are loud in their condemnation that he should so far, apparently, persist in keeping secret the nature of his alleged great discovery, and attribute to him unworthy motives in so doing. In this, we think, they do him great injustice. Koch has sense enough to know that if further time and experiment—both of which are necessary—shall prove that he has really made a disovery of the vast proportions indicated by the dispatches, he will not go unrewarded; that his nation will enrich him and load him with honors. Why then would he want to make money from the sale of his stuff? Without being in possession of any information on the subject further than that which has been made public, the JOURNAL submits as a reasonable solution of the question why he has not made public the details of his plan, that as yet it would be premature; he says himself, that he is not fully satisfied, and is daily experimenting, in the hope of being able in due time to announce positive results. To this end, it seems, he has given his secret and a portion of the "lymph" (which is said to be the remedy used) into the hands of a few reputable physicians for trial. Were he to allow every one calling himself "Doctor" to get hold of the lymph, no doubt great abuses would be made of it, and extortion practiced on the credulous sufferers; while discredit would be brought upon the whole thing.

As to the nature of his "cure," then, we can only speculate. Were it announced as a preventive instead of acure, one would naturally suppose, reasoning from analogy, and from what is known of vaccination as prophylactic of smallpox—and which would seem to furnish the key to the problem of preventing all diseases due to a living organism susceptible of cultivation—that he injects an attenuated cultivation of the tubercle bacillus; and in order that it should prevent consumption this would have to be done before the appearance of the bacillus in the patient; for such an inoculation would no more cure incipient phthisis than vaccination would cure small-pox. Hence, its range of application would be very limited, and the patients selected from those only in whom the disease is supposed to be hereditary; there would be no hope for the consumptive. But as it is not a preventive, but said to be a cure, we are driven to conclude that the lymph used can not be of the nature of a cultivation of the bacillus, but is simply a vehicle for the conveyance of a germicide, in some shape or form;—unless Koch can really exclaim "Eureka," and has found an antagonistic and more powerful bacillus than that of the tubercle,—one which will destroy the latter! But—would such a fellow—so deadly to the deadly tubercle microbe, be innocuous to the human system? That is the trouble with all germicides; in order that they may be fatal to the bacilli—they must be given in such strength as to be dangerous to human life. Were it not that corrosive sublimate will kill a man—it might be used to kill all the tubercle bacilli in existence. We venture the assurance that when Dr. Koch sees proper to make known the secret of his plan—it will be found to consist of fighting the tubercle bacillus *in situ* with some kind of a germicide.

The only thing to be apprehended is that the whole thing will blow over—and the great hopes and expectation now raised be blasted,—as was the case with Ferran's and Frier's discoveries; meantime, we can only wait and hope. It seems reasonable to hope that some day—prophylaxis of all diseases, caused by contagion, will be practicable—on the principle illustrated in vaccination against small-pox. We learn that in a short time Dr. Koch will be able to supply the lymph to responsible physicians for experiment, and at reasonable prices. So far, none has been brought to Texas—as will be seen from the extract from his letter published elsewhere.

If there is anything in it, what a valuable adjunct to the treatment can be found in the pure dry air in Southwest Texas; and at Austin, especially, a sanatarium should be established. Of this, more anon.

